# Ectopic Expression of the Grape Hyacinth (*Muscari armeniacum*) R2R3-MYB Transcription Factor Gene, *MaAN2*, Induces Anthocyanin Accumulation in Tobacco

**DOI:** 10.3389/fpls.2017.00965

**Published:** 2017-06-08

**Authors:** Kaili Chen, Hongli Liu, Qian Lou, Yali Liu

**Affiliations:** ^1^College of Landscape Architecture and Arts, Northwest A&F UniversityYangling, China; ^2^State Key Laboratory of Crop Stress Biology in Arid Areas, Northwest A&F UniversityYangling, China; ^3^College of Horticulture, Northwest A&F UniversityYangling, China

**Keywords:** flower color, monocots, grape hyacinth, R2R3-MYB transcription factor, anthocyanin biosynthesis

## Abstract

Anthocyanins are responsible for the different colors of ornamental plants. Grape hyacinth (*Muscari armeniacum*), a monocot plant with bulbous flowers, is popular for its fascinating blue color. In the present study, we functionally characterized an R2R3-MYB transcription factor gene *MaAN2* from *M. armeniacum*. Our results indicated that *MaAN2* participates in controlling anthocyanin biosynthesis. Sequence alignment and phylogenetic analysis suggested that MaAN2 belonged to the R2R3-MYB family AN2 subgroup. The anthocyanin accumulation of grape hyacinth flowers was positively correlated with the expression of *MaAN2*. And the transcriptional expression of *MaAN2* was also consistent with that of *M. armeniacum dihydroflavonol 4-reductase* (*MaDFR*) and *M. armeniacum anthocyanidin synthase* (*MaANS*) in flowers. A dual luciferase transient expression assay indicated that when MaAN2 was co-inflitrated with *Arabidopsis thaliana* TRANSPARENT TESTA8 (AtTT8), it strongly activated the promoters of *MaDFR* and *MaANS*, but not the promoters of *M. armeniacum chalcone synthase* (*MaCHS*), *M. armeniacum chalcone isomerase* (*MaCHI*), and *M. armeniacum flavanone 3-hydroxylase* (*MaF3H*). Bimolecular fluorescence complementation assay confirmed that MaAN2 interacted with AtTT8 *in vivo*. The ectopic expression of *MaAN2* in *Nicotiana tabacum* resulted in obvious red coloration of the leaves and much redder flowers. Almost all anthocyanin biosynthetic genes were remarkably upregulated in the leaves and flowers of the transgenic tobacco, and *NtAn1a* and *NtAn1b* (two basic helix–loop–helix anthocyanin regulatory genes) were highly expressed in the transformed leaves, compared to the empty vector transformants. Collectively, our results suggest that MaAN2 plays a role in anthocyanin biosynthesis.

## Introduction

Flowers, because of their varieties of colors, have great ornamental and economic value. In ornamental plant breeding, many measures are taken to breed different cultivars with various colors, hues, and patterns (Yamagishi et al., 2010). Pigmentation in flowers is mainly attributed to anthocyanins, a type of flavonoid; the major pigments imparting a great variety of colors from light yellow, red, dark red, magenta, purple, to blue in higher plants have been investigated extensively (Holton and Cornish, 1995; Winkel-Shirley, 2001; Hichri et al., 2011). Anthocyanin biosynthetic pathway is regulated by R2R3-MYB TFs and their interacting partners bHLH TFs and WD repeat or WD40 proteins (Zhang et al., 2014). Particularly, in the three different types of proteins, the R2R3-MYB TFs play crucial roles in anthocyanin biosynthesis and are usually considered more specific, because their expression influences the spatial and temporal distribution of anthocyanins (Holton and Cornish, 1995; Schwinn et al., 2006; Allan et al., 2008; Feller et al., 2011; Davies et al., 2012; Zhang et al., 2014).

The first R2R3-MYB C1 was discovered in the monocot *Zea mays*; it was involved in kernel pigmentation (Paz-Ares et al., 1987). Subsequently, in the dicot *Arabidopsis thaliana*, PRODUCTION OF ANTHOCYANIN PIGMENT1 (PAP1) and PAP2 were identified (Kranz et al., 1998); these proteins induced anthocyanin accumulation in *Arabidopsis* and *Nicotiana tabacum* (Borevitz et al., 2000). In dicot flowers, the anthocyanin-related R2R3-MYBs have been studied extensively and deeply in *Petunia hybrida* (Quattrocchio et al., 1999), *Antirrhinum majus* (Schwinn et al., 2006), *Ipomoea nil* (Morita et al., 2006), *Gerbera hybrida* (Elomaa et al., 2003; Laitinen et al., 2008), *Gentiana tricolor* (Nakatsuka et al., 2008a), and *Malus* crabapple (Jiang et al., 2014). Moreover, some studies have reported in monocot plants, such as *Elaeis guineensis* (Singh et al., 2014), *Anthurium andraeanum* (Li et al., 2016), *Lilium* (Yamagishi et al., 2010, 2011, 2014a,b; Yamagishi, 2011, 2015; Lai et al., 2012), *Allium cepa* (Schwinn et al., 2016), and Orchidaceae (Chiou and Yeh, 2008; Ma et al., 2009; Hsu et al., 2015; Li et al., 2017). In Araceae, *Anthurium andraeanum* AaMYB2 showed a positive correlation with pigmentation in anthurium spathes and leaves and induced anthocyanin accumulation in tobacco (Li et al., 2016). In Liliaceae, *Lilium* MYB6 regulated the development of anthocyanin spots in the tepals as well as the pigmentation in the leaves of the Asiatic hybrid lily ‘Montreux’ (Yamagishi et al., 2010), whereas MYB12 controlled the level of the coloration and pigmentation in the tepals of the Oriental and Asiatic hybrid lilies (Yamagishi et al., 2010, 2011, 2014a; Yamagishi, 2011, 2015; Lai et al., 2012). In Orchidaceae flowers, *Oncidium* ‘Gower Ramsey’ OgMYB1, produced red pigment when it was transiently overexpressed in the yellow lips (Chiou and Yeh, 2008). Purple *Phalaenopsis schilleriana* PsUMYB6 induced the production of anthocyanin when it was transiently co-expressed with Lc (a *Z. mays* bHLH TF determining anthocyanin accumulation) in the white *P. amabilis* petal tissue (Ma et al., 2009). PeMYB2, PeMYB11, and PeMYB12 regulated pigmentation in the petals and sepals, spots, and venation of red *P. equestris*, respectively (Hsu et al., 2015). Besides, *Dendrobium* hybrids DhMYB2 interacted with DhbHLH1 positively regulated pigmentation in the petals (Li et al., 2017).

Most R2R3-MYB TFs, as anthocyanin regulators in dicot flowers and a few monocots (oil palm, onion, anthurium, and lily), are included in the AN2 subgroup (corresponding to PhAN2 and AtPAP1 in dicots) (Yamagishi et al., 2010; Lai et al., 2012; Singh et al., 2014; Li et al., 2016; Schwinn et al., 2016). However, the anthocyanin-related MYBs in Orchidaceae are categorized into the C1 subgroup in Poaceae that includes *Z. mays* C1 and *Oryza sativa* MYB (CAA75509) (Chiou and Yeh, 2008; Ma et al., 2009; Hsu et al., 2015; Li et al., 2017). Schwinn et al. (2016) speculated that the distinct clades of AN2 and C1 subgroup in the R2R3-MYB family might occur within the monocot Asparagales in the evolution process of Allioideae and Orchidaceae. However, to date, only MYBs from Allioideae and Orchidaceae have been identified in the Asparagales (Chiou and Yeh, 2008; Ma et al., 2009; Hsu et al., 2015; Li et al., 2017). Therefore, more MYBs that control pigment synthesis should be studied in the monocots, especially in the Asparagales to address this issue.

Grape hyacinth (*Muscari armeniacum*), belonging to Scilloideae (Asparagales), is a monocot species used as cut flowers and garden ornamentals, and is famous for its fascinating blue color. To date, anthocyanin regulators in *M. armeniacum* have seldom been reported. In this study, we functionally identified an R2R3-MYB transcription factor MaAN2 from *M. armeniacum*, which plays a role in anthocyanin biosynthesis. And our findings might provide some information for the evolutionary divergence of R2R3-MYBs in monocots.

## Results

### Isolation of *MaAN2* from Grape Hyacinth Petals

**FIGURE 1:**
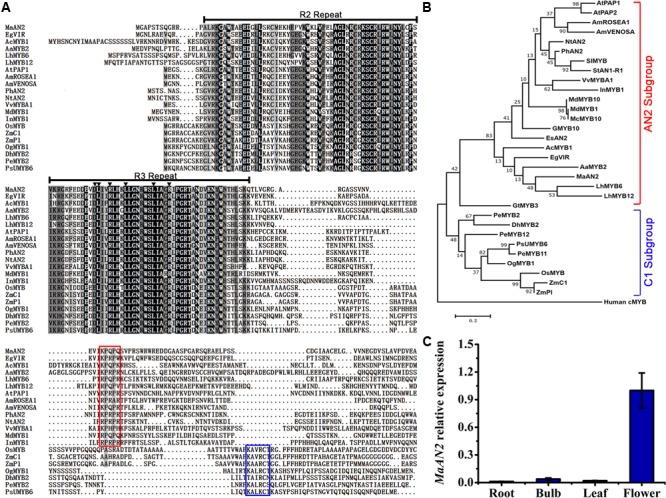
Protein sequence alignment and phylogenetic tree of MaAN2 transcription factor and its putative orthologs, and tissue-specific analysis of *MaAN2*. **(A)** Alignment of the deduced amino acid sequences of MaAN2 and other R2R3-MYBs associated with flavonoid biosynthesis from other plant species. If the identities of amino acids in each column are 100, 75, and 50%, these amino acids are indicated on a black, dark gray, and light gray background, respectively. The R2 and R3 repeats are indicated above the alignment. The motif [D/E]LX_2_[K/R]X_3_LX_6_LX_3_R in the R3 repeat necessary for interaction with a bHLH protein in the R3 repeat is indicated by dark triangles. The small motif [K/R]P[Q/R]P[Q/R] in the AN2 subgroup is shown by a red box. The short conserved sequences KAX[K/R]C[S/T], recognized in the C1 subgroup, are shown by blue box. **(B)** The maximum-likelihood phylogenetic tree was generated using MEGA 6.0 software. Numbers next to the nodes indicate the bootstrap values from 1000 replications. The bar indicates a genetic distance of 0.1. The gene names from various plant species and NCBI GenBank accession numbers for the sequences are as follows: *Arabidopsis thaliana* AtPAP1 (NP_176057), AtPAP2 (NP_176813); *Petunia hybrida* PhAN2 (AAF66727); *Nicotiana tabacum* NtAN2 (ACO52472); *Solanum lycopersicum* SlMYB (AAQ55181); *Antirrhinum majus* AmROSEA1 (ABB83826), AmVENOSA (ABB83828)); *Ipomoea nil* InMYB1 (BAE94391.1); *Gerbera hybrida* GMYB10 (CAD87010); *Vitis vinifera* VvMYBA1 (BAD18977); *Malus domestica* MdMYB1 (ABK58138) and MdMYB10 (ABB84753); *Malus* crabapple McMYB10 (AFP89357); *Epimedium sagittatum* EsAN2 (ALO24362); *Solanum tuberosum* StAN1-R1 (AKA95392); *Elaeis guineensis* EgVIR (KJ789862); *Allium cepa* AcMYB1 (KX785130); *Anthurium andraeanum* AaMYB2 (KU726561); *Lilium hybrid* division I LhMYB6 (BAJ05399) and LhMYB12 (BAJ05398); *Gentiana triflora* GtMYB3 (BAF96933); *Oncidium* Gower Ramsey OgMYB1 (ABS58501); *Zea mays* ZmPl (NP_001105885) and ZmC1 (NP_001106010); *Oryza sativa* OsMYB (CAA75509); *Phalaenopsis schilleriana* PsUMYB6 (ACH95792); *Phalaenopsis equestris* PeMYB2 (AIS35919), PeMYB11 (AIS35928), and PeMYB12 (AIS35929); *Dendrobium hybrid* DhMYB2 (AQS79852); Human cMYB (M15024). **(C)** The expression profile of *MaAN2* in the roots, bulbs, leaves, and flowers of *Muscari armeniacum.*

An anthocyanin-related *R2R3-MYB* unigene was screened from the transcriptome of *M. armeniacum* flowers (Lou et al., 2014). The 723-bp full-length *MaAN2* cDNA encoding 240 amino acids was isolated from the flowers of *M. armeniacum* by using the RACE method. An amino acid alignment of R2R3-MYBs in grape hyacinth and other species showed that the R2 and R3 repeats were highly conserved in the N-terminal of MaAN2 (**Figure 1A**). However, when considering the entire protein sequence, MaAN2 showed only 48.12% identity with EgVIR (*Elaeis guineensis* anthocyanin-related R2R3-MYB), 39.92% identity with PhAN2, 39.12% identity with LhMYB6, and 33.55% identity with AaMYB2. Besides, in the R3 repeat, a conserved motif [D/E]LX_2_[R/K]X_3_LX_6_LX_3_R (as marked by black triangles in **Figure 1A**) is necessary for its interaction with the R-like bHLH protein (Zimmermann et al., 2004; Takos et al., 2006). Like LhMYB6 and LhMYB12 in *Lilium*, MaAN2 protein also had a conserved motif [K/R]P[Q/R]P[Q/R] in the C-terminal variable region (as marked by red box in **Figure 1A**), which was responsible for anthocyanin biosynthesis in eudicots (Yamagishi et al., 2010).

The phylogenetic analysis of MaAN2 and other R2R3-MYBs from different species showed that MaAN2 was clustered closely with the sequences of monocots AaMYB2 and LhMYB6 in the AN2 subgroup, while the MYBs from monocot Poaceae and Orchidaceae were in C1 subgroup (**Figure 1B**). In addition, the transcription expression of *MaAN2* in roots, bulbs, leaves, and flowers were analyzed by Quantitative Real-Time PCR (qRT-PCR) assay. The result showed that the expression of *MaAN2* was very low in roots, bulbs, and leaves, but very high in flowers, which indicated that *MaAN2* was predominantly expressed in flowers (**Figure 1C**).

### MaAN2 Is a Transcription Factor

**FIGURE 2:**
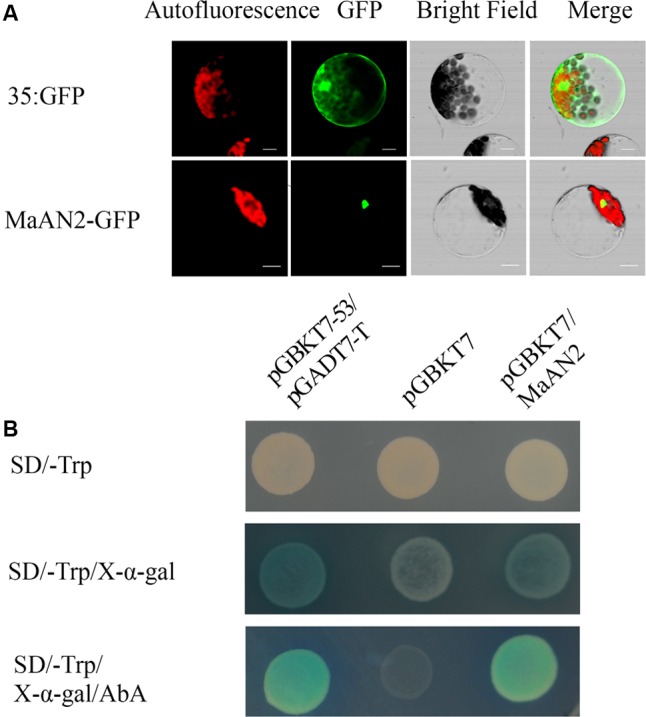
Subcellular localization and transcription activation ability of MaAN2. **(A)** Transient expression of the MaAN2-GFP fusion protein in *A. thaliana* mesophyll protoplasts showed MaAN2 located in the nucleus. Autofluorescence: chloroplast autofluorescence; GFP: GFP fluorescence; Merge is merged with chloroplast autofluorescence, GFP fluorescence, and bright field images. Bars, 10 μm. **(B)** Transcription activation ability of MaAN2 in Y2Hgold yeast. The positive control was pGBKT7-53 plus pGADT7-T and the negative control was pGBKT7. Yeasts transformed with the positive and negative controls, and pGBKT7-MaAN2 vectors were cultivated in SD/-Trp medium, SD/-Trp medium with 40 μg/ml X-α-Gal, and SD/-Trp medium plus 40 μg/ml X-α-Gal and 200 ng/ml AbA, respectively. The positive control and pGBKT7-MaAN2 exhibited blue yeast plaques, while the negative control did not grow in SD/-Trp medium plus 40 μg/ml X-α-Gal and 200 ng/ml AbA.

In order to detect whether MaAN2 is located in cell nucleus, the plasmid pCambia1304-MaAN2 (MaAN2-GFP) was transformed into *Arabidopsis* mesophyll protoplasts, and the nuclear localization of MaAN2 in *Arabidopsis* protoplasts was thus confirmed. In the positive control 35S:GFP (pCambia1304), the expression of GFP was found to be distributed throughout the entire cell (**Figure 2A**).

To determine whether MaAN2 has the transcriptional activity, we conducted a transactivation assay in yeast. Yeasts transformed with pGBKT7-MaAN2 vector and the positive control pGBKT7-53 plus pGADT7-T exhibited blue yeast plaques, while the negative control pGBKT7 did not grow in SD/-Trp medium plus 40 μg/ml X-α-Gal and 200 ng/ml AbA (**Figure 2B**). These results showed that MaAN2 possessed transcriptional activity. Therefore, MaAN2 was thought to be a transcription factor.

### *MaAN2* Expression Correlates with Anthocyanin Biosynthetic Gene Expression and Anthocyanin Accumulation during Grape Hyacinth Flowering

**FIGURE 3:**
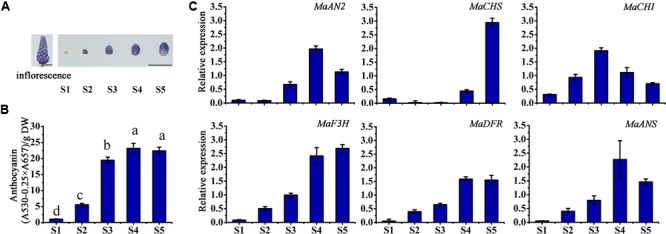
The anthocyanin content and expression profiles of anthocyanin regulatory and structural genes at different flower developmental stages in *M. armeniacum*. **(A)** The inflorescence and the petals of five flower developmental stages of *M. armeniacum*. Bars, 1 cm. **(B)** The total anthocyanin content of petals at the five flower developmental stages. DW: dry weight. Different lower case letters represent significant difference calculated using Least–Significant Difference (*LSD*) analysis at the level of *P <* 0.05. **(C)** The expression profiles of *MaAN2, MaCHS, MaCHI, MaF3H, MaDFR*, and *MaANS* in flowers during the five developmental stages (S1–S5) of *M. armeniacum*. *MaActin* was the reference gene to normalize the expression of these genes. Each column represents means ± SD from three independent experiments.

The inflorescence and the petals of different flower developmental stages (S1–S5) of *M. armeniacum* are shown in **Figure 3A**. The total anthocyanin content of *M. armeniacum* at the five flowering stages was significantly different. The anthocyanin content first increased, and then decreased, during the flower development period, peaking at stage S4 (**Figure 3B**). Specifically, the anthocyanin was seldom detected at stage S1, and accumulated at stage S2. The anthocyanin content showed a rapid increase from stage S2 to S4, but decreased from stage S4 to S5.

In order to detect the expression levels of anthocyanin biosynthetic genes during flower development, the anthocyanin biosynthetic genes: *chalcone synthase* (*CHS*), *chalcone isomerase* (*CHI*), *flavanone 3-hydroxylase* (*F3H*), and *anthocyanidin synthase* (*ANS*) (as markedby red color in Supplementary Figures S1–S4), were screened from the *M. armeniacum* flower transcriptome (Lou et al., 2014), as well as *M. armeniacum dihydroflavonol 4-reductase* (*MaDFR*; Jiao et al., 2014) were used in this study. And then, the transcription levels of *MaAN2* and pigment biosynthetic genes were analyzed using the qRT-PCR for stage S1–S5 (**Figure 3C**). The transcriptional expression of *MaAN2* was correlated with that of *MaDFR* and *MaANS* (**Figure 3C**). The expression levels of *MaDFR, MaANS*, and *MaAN2* first increased and peaked at stage S4, and then decreased with flower development; this expression pattern was similar to the anthocyanin content changes during progression from stage S1 to S5 (**Figures 3B,C**). Furthermore, the mRNA levels of *MaCHS* and *MaCHI* were not correlated with anthocyanin biosynthesis during flower blossoming. *MaF3H* expression increased from stages S1 to S4, coordinating with that of *MaAN2*, but the expression of *MaF3H* continued to increase until stage S5.

### MaAN2 Interacts with a bHLH Protein to Activate the Promoters of Anthocyanin Structural Genes

**FIGURE 4:**
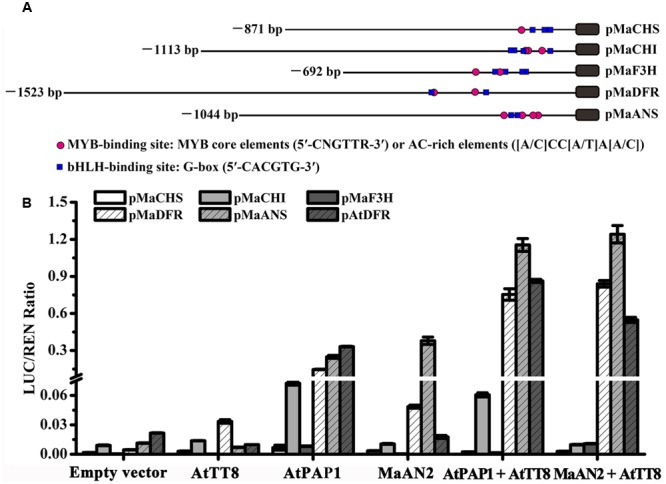
Schematics of *M. armeniacum* anthocyanin biosynthetic gene promoters and dual luciferase transient expression assay. **(A)** The lengths, MYB binding sites, and bHLH binding sites of *MaCHS, MaCHI, MaF3H, MaDFR*, and *MaANS* promoters are marked by black solid lines, red circles, and blue rectangles, respectively. **(B)** Dual luciferase transient expression assay showed the effects of MaAN2, AtPAP1, and AtTT8 TFs on *MaCHS, MaCHI, MaF3H, MaDFR, MaANS*, and *A. thaliana AtDFR* promoters. All TFs were co-infiltrated with promoters in *N. benthamiana* leaves for transient transformation assays. The results show the promoter activity expressed as the ratio of the activities of these promoters: luciferase (LUC) to 35S: Renilla (REN). Each column represents means ± SD from four biological replicates.

To investigate whether MaAN2 could interact with the bHLH TF to regulate the expression of anthocyanin pathway genes, the different TFs and promoters were co-infiltrated in *N. benthamiana* leaves, and subsequently, a dual-luciferase assay was conducted (**Figure 4**). Firstly, we cloned 871, 1113, 692, and 1044 bp of promoters of anthocyanin biosynthetic genes (*MaCHS, MaCHI, MaF3H*, and *MaANS*; as marked by red color in Supplementary Figures S1–S4), respectively; these genes were screened from the *M. armeniacum* flower transcriptome (Lou et al., 2014). Moreover, we also cloned 1523 bp promoter of *MaDFR* (Jiao et al., 2014). Next, we analyzed the *cis*-elements of these gene promoters (**Figure 4A**) using the online website PlantCARE. All promoters contained the MYB-binding sites: MYB core element (5′-CNGTTR-3′) or the AC-rich element ([A/C]CC[A/T]A[A/C]) (Xu et al., 2015) and the bHLH-binding site: G-box (5′-CACGTG-3′) (Hichri et al., 2011) (**Figure 4A**). Thus, these genes might be regulated by MYB and bHLH TFs.

Further, the R2R3-MYB protein PAP1 and bHLH protein TT8 involved in the regulation of flavonoid synthesis in *Arabidopsis* (Nesi et al., 2000) along with the *AtDFR* promoter were cloned and used as positive control. The result showed that the promoter activities of *MaDFR, MaANS*, and *AtDFR* indicated a considerable increase when MaAN2 or AtPAP1 was co-infiltrated with AtTT8, compared with the treatment with MYBs alone (**Figure 4B**). The promoter activities of *MaCHS, MaCHI*, and *MaF3H* were very low when MaAN2 or AtPAP1 co-infiltrated with or without AtTT8 (**Figure 4B**). MaAN2 could not promote the expression of *MaCHS, MaCHI, MaF3H, MaDFR*, and *AtDFR*, but could slightly activate the *MaANS* promoter without the co-expression of AtTT8. In brief, MaAN2 depended on the presence of bHLH protein AtTT8 to activate the expression of *MaDFR, MaANS*, and *AtDFR*.

**FIGURE 5:**
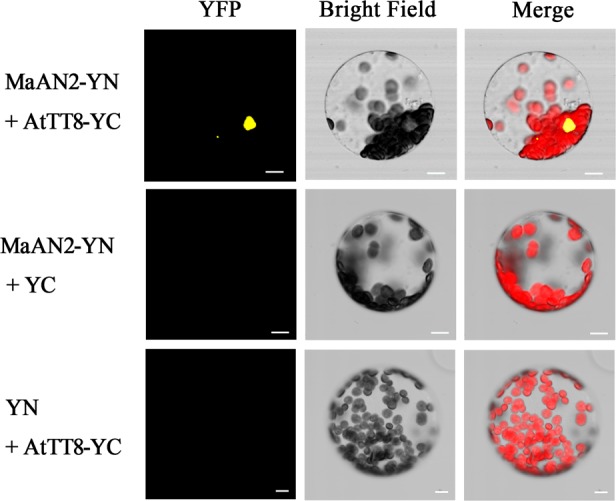
Bimolecular fluorescence complementation of MaAN2 and AtTT8 interaction in *A. thaliana* mesophyll protoplasts. YFP: fluorescence of YFP; Merge is digital image merged with bright field and fluorescent images. Bars, 10 μm.

In order to confirm the interaction between MaAN2 and AtTT8 in *vivo*, a BiFC assay was conducted in *A. thaliana* mesophyll protoplasts (**Figure 5**). The YFP expression was detected only when pSPYNE/MaAN2 was co-expressed with pSPYCE/AtTT8, but not when pSPYNE/MaAN2 was co-expressed with pSPYCE and pSPYCE/AtTT8 was co-expressed with pSPYNE. Thus, MaAN2 interacted with bHLH TF AtTT8 *in vivo*.

### Overexpression of *MaAN2* Promotes Anthocyanin Accumulation in Tobacco

**FIGURE 6:**
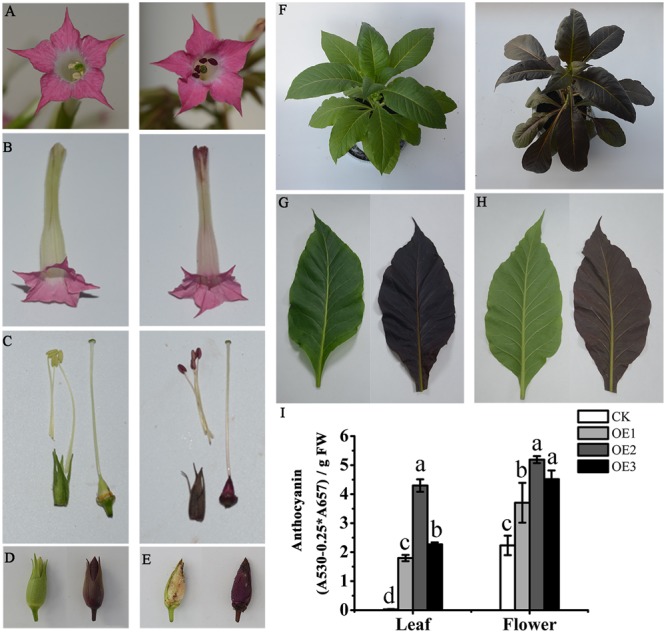
Phenotypic observation and anthocyanin content in empty vector (CK) and *OE*-*MaAN2* tobacco transplants. The front **(A)** and lateral **(B)** parts of flower; anthers, stigma, and ovary **(C)**; the sepal **(C,D);** and seeds **(E)**, total plant **(F)**, the adaxial side **(G)** and abaxial side **(H)** of a leaf. The left sides in each block show the transgenic tobacco of CK, whereas the right sides in each block show the transgenic tobacco of *OE*-MaAN2 (i.e., OE2). **(I)** Total anthocyanin content of the leaf and flower of CK and three independent *OE*-MaAN2 transgenic lines (OE1, OE2, and OE3). Each column represents means ± SD from three independent experiments. Different lower case letters represent significant difference which is calculated using *LSD* analysis at the level of *P <* 0.05.

To characterize the function of *MaAN2*, we obtained the overexpressing *MaAN2* transgenic tobaccos (OE-*MaAN2*) by the method of tobacco leaf disks. The result showed that *MaAN2* promoted pigmentation in the corolla of flowers (**Figures 6A,B**). Besides, the pistil, anther, sepal, ovary, and seeds showed the strong accumulation of anthocyanins (**Figures 6C–E**); the whole tobacco leaves of OE-*MaAN2* turned red (**Figures 6F–H**).

Additionally, in the leaves and flowers of three OE-*MaAN2* lines (OE1, OE2, and OE3), the total anthocyanin contents were significantly increased compared to those in the empty vector transgenic tobaccos (CK) (**Figure 6I**). Next, we used HPLC to analyze the anthocyanin composition in the transgenic tobacco leaves and corollas. Previous studies reported that cyanidin-3-rutinoside mainly existed in the petal of wild-type tobacco (Deluc et al., 2006; Nakatsuka et al., 2008b). Even in our study, cyanidin-3-rutinoside was mainly detected in the leaves and flowers of OE-*MaAN2* (Supplementary Figure S5).

### Overexpression of *MaAN2* Promotes the Expression of Anthocyanin Pathway Genes in Tobacco

**FIGURE 7:**
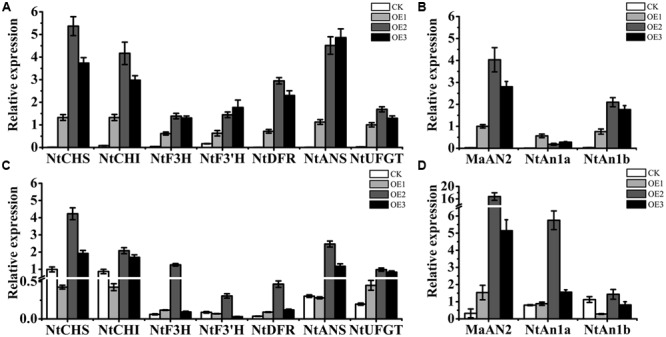
Expression profiles of anthocyanin biosynthetic genes and endogenous transcription factors in the leaves and flowers of the empty vector transgenic tobaccos (CK) and three independent *OE*-MaAN2 transplants (OE1, OE2, and OE3). Expression patterns of *NtCHS, NtCHI, NtF3H, NtF3′H, NtDFR, NtANS*, and *NtUFGT* genes in the leaves **(A)** and flowers **(C)** of CK and OE1, OE2, and OE3. Expression patterns of *MaAN2, NtAn1a*, and *NtAn1b* in the leaves **(B)** and flowers **(D)** of CK and OE1, OE2, and OE3. *NtTubA1* was the reference gene to normalize the expression of these genes. Each column represents means ± SD from three independent experiments.

By qRT-PCR assay, the presence of *MaAN2* was confirmed in transgenic tobacco (**Figures 7B,D**). We found that *MaAN2* positively regulated the transcriptional expression of anthocyanin biosynthetic genes in tobacco leaves and flowers. The anthocyanin biosynthetic genes, *NtCHS, NtCHI, NtF3H, NtF3′H, NtDFR, NtANS*, and *NtUFGT*, were upregulated in the leaves of OE1, OE2, and OE3 (**Figure 7A**). Specifically, the transcripts of *NtCHS, NtCHI*, and *NtANS* were remarkably increased in the three transgenic lines, especially in OE2 and OE3. In the flowers of transgenic tobacco, the expressions of *NtCHS, NtCHI, NtF3H, NtF3’H, NtDFR, NtANS*, and *NtUFGT*, were increased in OE2 (**Figure 7C**). However, in the flowers of OE1, the expressions of *NtCHS, NtCHI, NtF3’H*, and *NtANS* were slightly decreased, and the expressions of the other pigment genes maintained or slightly increased compared to that of CK (**Figure 7C**), which might be because the expression of *MaAN2* in OE1 was much weaker than that in OE2 and OE3 (**Figure 7D**). The endogenous *bHLH* TF genes, *NtAn1a* and *NtAn1b*, homologous to *AtTT8*, which regulated anthocyanin biosynthesis in tobacco (Bai et al., 2011), were upregulated in the leaves of OE1, OE2, and OE3 (**Figure 7B**). Further, the transcription level of *NtAn1a* in the transplant flowers was considerably higher in OE2 than that in CK (**Figure 7D**). However, the transcription level of *NtAn1b* in transgenic flowers was similar to that in CK (**Figure 7D**).

## Discussion

### MaAN2 Belongs to the AN2 Subgroup

R2R3-MYBs play vital roles in plant anthocyanin biosynthesis; and they usually have a conserved DNA-binding domain (R2 and R3 repeats) in the N-terminal and a variable C-terminal region (Dubos et al., 2010). In dicots, the [K/R]P[Q/R]P[Q/R] motif in the C-terminal is the typically characteristic of the AN2 subgroup in R2R3-MYB TFs family (Yamagishi et al., 2010). MaAN2, like other monocot MYBs, such as LhMYBs, EgVIR, AaMYB2, and AcMYB1 (*Allium cepa* anthocyanin activator), contained the motif [K/R]P[Q/R]P[Q/R] (**Figure 1A**), not the conserved motif KAx[K/R]C[S/T] (as marked by blue box in **Figure 1A**), which is the characteristic of the C1 subgroup and usually appears in monocot maize (Cone et al., 1993).

*Muscari armeniacum* belongs to the Scilloideae subfamily, which is included in the family Asparagaceae of the order Asparagales (Angiosperm Phylogeny Group III, 2009). The anthocyanin-related MYBs have also been studied in the family Orchidaceae (*Oncidium, Phalaenopsis*, and *Dendrobium*) (Chiou and Yeh, 2008; Ma et al., 2009; Hsu et al., 2015; Li et al., 2017) and Allioideae (*Allium cepa*) (Schwinn et al., 2016), which also belong to the order Asparagales (Angiosperm Phylogeny Group III, 2009). Besides, some other anthocyanin MYB regulators have been reported in the monocots, including Arecaceae (*E. guineensis*; Singh et al., 2014), Araceae (*Anthurium andraeanum*; Li et al., 2016), and Liliaceae (*Lilium*; Yamagishi et al., 2010), as well as Poaceae (*Z. mays*; Paz-Ares et al., 1987). In these reported MYBs in monocots, the MYBs from Orchidaceae and Poaceae were clustered in the C1 subgroup, and the others were included in the AN2 subgroup. Hence, the anthocyanin-related MYBs showed divergence among the monocot species. However, they are conserved in the reported dicot species (Davies et al., 2012).

### MaAN2 Interacts with a bHLH Protein AtTT8 and Targets *MaDFR* and *MaANS* to Control Anthocyanin Pigmentation

In the flower development of grape hyacinth, the expression profile of *MaAN2*, as well as those of *MaDFR* and *MaANS*, was positively concomitant with the flower pigmentation in *M. armeniacum* (**Figure 3**). Furthermore, via the dual luciferase transient expression assay, we found that MaAN2 was highly dependent on AtTT8 as a partner to activate the promoters of *MaDFR* and *MaANS* (**Figure 4B**), and the BiFC assay also confirmed that MaAN2 interacted with AtTT8 *in vivo* (**Figure 5**). Additionally, the expression profile of *MaF3H* was similar to those of *MaDFR, MaANS*, and *MaAN2* in flowers at stages S1–S4 (**Figure 3C**), but it was not activated by MaAN2 (**Figure 4B**). In plant, anthocyanin biosynthesis is mainly catalyzed by a set of enzymes encoded by anthocyanin structural genes (Dooner et al., 1991; Grotewold, 2006; Katsumoto et al., 2007; Tanaka et al., 2008); these structural genes are usually classified into early and late biosynthetic genes (EBGs and LBGs) depending on different species (Martin et al., 1991; Quattrocchio et al., 1993; Boss et al., 1996; Pelletier et al., 1997; Gonzalez et al., 2008). In *Z. mays*, the EBGs and LBGs are usually regulated by the MYB-bHLH (i.e., ZmC1-ZmLc) complex (Dooner et al., 1991). However, in dicot *Arabidopsis*, these genes are controlled separately by different TFs (Dubos et al., 2010); LBGs are regulated by the complex of AtPAP1-AtTT8 (Xu et al., 2014). PhAN2 interaction with the bHLH PhAN1 is able to activate the *PhDFR* promoter (Spelt et al., 2000). GtMYB3 interaction with GtbHLH1 could activate the expressions of LBGs: flavonoid 3′, 5′-hydroxylase (*F3*′*5*′*H*) and hydroxycinnamoyl-CoA: anthocyanin 5-*O*-acyltransferase (*5AT*) (Nakatsuka et al., 2008a). In *M. armeniacum, MaF3H* might be an EBG; its expression was not activated by MaAN2 but possibly by some other TF(s). In *Arabidopsis, F3H*, as the EBG, is regulated by subgroup 7 R2R3-MYBs (Dubos et al., 2010). Moreover, in *Antirrhinum majus, Lilium* spp., and *Phalaenopsis* spp., there are two or three MYBs responsible for their flower pigmentation (Schwinn et al., 2006; Yamagishi et al., 2010; Hsu et al., 2015). In the present study, *MaAN2* expression at stage S2 was similar to that at stage S1, but the expression levels of *MaDFR* and *MaANS* were higher at stage S2 than at stage S1 (**Figure 3C**), which indicated that the likely involvement of other TFs in flower pigmentation. In brief, our study suggested that MaAN2 interacted with AtTT8 to control LBGs (*MaDFR* and *MaANS*) expression. However, in *Lilium* spp., LhMYB12 activated *LhCHSa* (EBG) and *LhDFR* (LBG) expression in the presence of LhbHLH2 in Asiatic hybrid lily tepals (Lai et al., 2012). In *Phalaenopsis* spp., PeMYBs could activate the expression of *F3H5* (EBG), *DFR1* and *ANS3* (LBGs) in flowers to influence the patterns and quantity of color. When *Oncidium* ‘Gower Ramsey’ *OgMYB1* was overexpressed in its yellow lip, the transcripts of *OgCHI* and *OgDFR* were increased (Chiou and Yeh, 2008).

Overall, the regulation pattern of anthocyanin-related MYBs is conserved in dicots (i.e., the interaction of MYB with bHLH-activating LBGs) (Dubos et al., 2010; Xu et al., 2014, 2015; Li et al., 2017). Moreover, we proposed that the anthocyanin-related MYBs originating from the monocots except Poaceae and Orchidaceae were included in the AN2 subgroup and depended on the bHLH TFs for inducing anthocyanin biosynthesis. However, R2R3-MYBs from Gramineae and Orchidaceae belong to the C1 subgroup (Hsu et al., 2015; Li et al., 2017). Whether these MYBs regulated EBGs and LBGs or only LBGs varies with different species in monocots (Chiou and Yeh, 2008; Lai et al., 2012; Hsu et al., 2015). Further, whether the distinction in activating EBGs or LBGs is caused by the MYB subgroups or by the divergence between monocots and dicots is not yet known (Lai et al., 2012), thus more evidence is needed to be provided for finding the regularity.

### MaAN2 Promotes Anthocyanin Accumulation by Upregulating the Expression of Anthocyanin Biosynthetic and *bHLH* TF Genes

Many studies showed that ectopically expressing *R2R3-MYB* genes of the AN2 subgroup, such as *AtPAP1* and *AtPAP2* (Borevitz et al., 2000; Xie et al., 2006), *Epimedium sagittatum EsAN2* (Huang et al., 2016b), *Solanum tuberosum StAN1-R1* (Liu et al., 2016), *AaMYB2* (Li et al., 2016), and *LhMYB12-Lat* (Yamagishi et al., 2014b) could regulate anthocyanin biosynthesis to various extent in tobacco. In this study, the OE-*MaAN2* accumulated anthocyanin in the flowers and leaves and showed distinct phenotypes compared with CK (**Figures 6A–H**). The anthocyanin contents were significantly higher in the pigmented leaves and flowers of OE-*MaAN2* than in those of CK (**Figure 6I**).

Zhang et al. (2014) considered that both MYB and bHLH partners can likely induce the entire biosynthetic pathway for anthocyanin accumulation. Our study showed that the overexpression of *MaAN2* in tobacco could differentially activate the expression of anthocyanin biosynthetic genes. The EBGs (*NtCHS, NtCHI*, and *NtF3H*) as well as the LBGs (*NtDFR, NtANS*, and *NtUFGT*) were activated in leaves of three transgenic lines and flowers of OE2 and OE3 (**Figures 7A,C**). This result is consistent with that of overexpression of *StAN1-R1* (a potato R2R3-MYB anthocyanin regulator) in tobacco (Liu et al., 2016); the result was also observed in the leaves of *N. tabacum* ectopically expressing *EsAN2* or *AaMYB2* (Huang et al., 2016b; Li et al., 2016). Two bHLH TF genes, *NtAn1a* and *NtAn1b*, controlled anthocyanin biosynthesis in the corollas of tobacco plants (Bai et al., 2011); they were also activated in the transgenic leaves when ectopically expressing *EsAN2, StAN1-R1*, or *AaMYB2* in tobacco (Huang et al., 2016b; Li et al., 2016; Liu et al., 2016). The similar result was also found in the leaves of OE-*MaAN2* tobacco (**Figure 7B**). However, in flowers, the transcripts of *NtAn1a* and *NtAn1b* were not influenced by EsAN2 (Huang et al., 2016b), but strongly upregulated by StAN1-R1 (Liu et al., 2016). In this study, *MaAN2* activated the expression of *NtAn1a*, not *NtAn1b* in transplant flowers (**Figure 7D**). Therefore, the *bHLH* gene expression activated by MYBs were different between leaves and flowers in tobacco. Overall, the heterogeneous R2R3-MYBs could activate the expression of endogenous *bHLH* genes in *N. tabacum* leaves. Then these AN2 subgroup MYBs might interact with the endogenous bHLH TF to enhance anthocyanin contents. However, in the tobacco leaves of overexpressing *LhMYB12-Lat* (an anthocyanin regulatory *R2R3-MYB* gene), it is showed higher levels of the transcripts of *NtCHS, NtCHI, NtF3H, NtDFR*, and *NtANS*. Whereas, LhMYB12-Lat could not activate the transcription of *bHLH* in a heterologous system (Yamagishi et al., 2014b). Therefore, MYBs from different species might exert diverse effects.

## Conclusion

The newly identified R2R3-MYB MaAN2 was possibly an anthocyanin activator involved in grape hyacinth flower coloration. It belongs to the AN2 subgroup of R2R3-MYB family, and interacts with a bHLH to regulate the expression of LBGs. The regulatory pattern of anthocyanin biosynthesis is similar to that in dicot. The finding might provide a sight into elucidating of the anthocyanin biosynthesis in grape hyacinth and suggest the evidence for the evolutionary divergence of R2R3-MYB TFs in monocot lineages.

## Materials and Methods

### Plants Materials

Plants of grape hyacinth *M. armeniacum* were cultivated in an experimental field of the Northwest A&F University in the Yangling District of the Shaanxi Province in China. The flower development is divided into five stages mainly based on petal pigmentation: S1, no pigmentation; S2, pigmentation visible on the basal part; S3, pigmentation beginning to turn blue; S4, flowers were completely blue, but had not opened; and S5, flowers completely opened. Besides, the roots, bulbs, and leaves of *M. armeniacum* were reserved. *N. tabacum* ‘NC89’ was cultured in a light incubator with a 16/8 h day/night and used for genetic transformation at the four-leaf stage; the transplant tobaccos were transferred from aseptic culture room to a greenhouse in natural light and artificial light extension to 16 h. The fully expanded tobacco leaves were collected from the mature transplants, and the flower limbs were picked when the corollas opened at an angle of 90°. All samples collected from grape hyacinth and tobacco were frozen in liquid nitrogen and stored at -80°C.

### qRT-PCR Assay

Total RNA was isolated from *M. armeniacum* flowers, roots, bulbs, and leaves as well as from the leaves and flowers of tobacco using the Omega Total RNA Kit (Omega, United States). For this, 1 μg RNA aliquots were treated with the PrimeScript RT reagent Kit with gDNA Eraser (Takara, Japan) to remove the residual genomic DNA and then synthesize cDNA. The cDNA was diluted five times and used as the template, and the BioEasy Master Mix Plus (SYBR Green) (Bioer, China) was used as the fluorochrome for qPCR assay. Specifically, the cDNA templates from five flower stages were mixed equally and used for qRT-PCR analysis to detect the tissue-specific expression of *MaAN2*. The assay was conducted by using the iQ5 RT-PCR detection system (Bio-Rad, United States). The qRT-PCR primers of grape hyacinth and tobacco are listed in Supplementary Table S1. *MaActin* and *NtTubA1* were used as the internal control genes in each grape hyacinth and tobacco sample, respectively. All analyses were conducted with three independent experiments.

### Isolation of Full-Length Coding Sequence of *MaAN2*

We obtained an anthocyanin-related *R2R3-MYB* unigene from the transcriptome of *M. armeniacum* flowers (Lou et al., 2014). This DNA fragment lacked the 5′- and 3′-untranslated regions; thus, we isolated full-length cDNA of *MaAN2* from the flowers in *M. armeniacum* followed the method described by Huang et al. (2016b). The primers used for the 5′- and 3′-RACE PCR, as well as full-length gene-specific primers are listed in Supplementary Table S2. Finally, this cDNA sequence was submitted to the NCBI GenBank database with an accession number KY781168. Besides, the R2R3-MYB was clustered with the AN2 subgroup; therefore, it was considered as *MaAN2*.

Multiple alignments were analyzed using DNAMAN 8.0. The R2 and R3 repeats as well as the two conserved motifs were highlighted with differently colored lines and boxes. The listed R2R3-MYBs were downloaded from the GenBank database (accession numbers listed in **Figure 1**). The program MEGA 6.0 was used for constructing a phylogenetic tree by maximum likelihood method.

### Subcellular Localization of MaAN2

For subcellular localization analysis, the ORF of *MaAN2* was introduced into the pCambia1304 vector using the Seamless Cloning and Assembly Kit (Novoprotein, Shanghai; primers are listed in Supplementary Table S3). The C-terminal of MaAN2 protein was fused to GFP, which allowed the transient expression of these proteins *in planta*. The polyethylene glycol-mediated transfection of *A. thaliana* mesophyll protoplasts was conducted according to previously described protocols (Yoo et al., 2007). 35S:GFP (pCambia1304) alone (positive control), chlorophyll fluorescence and MaAN2-GFP (pCambia1304-MaAN2) were observed 16 h after transformation using a FV1000 confocal microscope (Olympus, Japan). Images were analyzed using the FV1000 Viewer software.

### Transcription Activation Ability of MaAN2

To determine the transcription activation ability of MaAN2, a yeast expression vector was constructed by fusing the ORF PCR product into a pGBKT7 vector using the Seamless Cloning and Assembly Kit (Novoprotein, Shanghai; primers are listed in Supplementary Table S3). The vectors pGBKT7-53 plus pGADT7-T (positive control), pGBKT7 (negative control), and pGBKT7-MaAN2 were introduced into the yeast strain Y2Hgold according to the Yeastmaker^TM^ Yeast Transformation System 2 User Manual (Clontech, Japan). These transformants were cultivated at 30°C for about 3 d on SD/-Trp medium, SD/-Trp medium with 40 μg/ml X-α-Gal, and SD/-Trp medium plus 40 μg/ml X-α-Gal and 200 ng/ml AbA.

### Isolation of *MaCHS, MaCHI, MaF3H, MaDFR*, and *MaANS* Promoters by Genome Walking

In order to explore whether MaAN2 regulates the expression of anthocyanin biosynthetic genes, the promoters of anthocyanin biosynthetic genes were needed to clone. We screened the unigenes of each anthocyanin biosynthetic gene from the *M. armeniacum* flower transcriptome (Lou et al., 2014), including *MaCHS, MaCHI, MaF3H*, and *MaANS.* The unigene of each anthocyanin biosynthetic gene which showed higher expression among its respective candidate genes and meanwhile could be clustered with its respective orthologs was used in this study (as marked by red color in Supplementary Figures S1–S4). Then these anthocyanin biosynthetic genes used in this study were submitted to NCBI GenBank database. The accession numbers of *MaCHS, MaCHI, MaF3H*, and *MaANS* were MF041820, MF041821, MF041822, and MF041819, respectively. Notably, the cDNA of *M. armeniacum DFR* (KJ619963) reported by Jiao et al. (2014) also used in this study. The promoter regions of these genes were cloned from *M. armeniacum* genomic DNA by conducting a nested PCR with the genome walker kit (Clontech, United States). The nested PCR assays were performed using the degenerate primer from the kit and three gene-specific primers, as described in Supplementary Table S4. The obtained sequences were analyzed using DNAMAN 8.0. Finally, we isolated 871, 1113, 692, 1523, and 1044 bp promoter sequences of *MaCHS, MaCHI, MaF3H, MaDFR*, and *MaANS*, with accession numbers KY781171, KY781172, KY781173, KY781169, KY781170 in NCBI GenBank database, respectively. In addition, the *AtDFR* (AT5G42800) promoter sequence, which was used as the positive control, was downloaded from TAIR^1^ and obtained from the *Arabidopsis* genomic DNA. The *cis*-elements of these promoters were speculated using the online website PlantCARE^2^.

### Dual Luciferase Assay

For dual luciferase assay, the full-length sequences of TF genes: *MaAN2, AtPAP1* (AT1G56650), *AtTT8* (AT4G09820), and the promoters: *MaCHS, MaCHI, MaF3H, MaDFR, MaANS*, and *AtDFR*, were amplified using primers listed in Supplementary Table S5, and jointed with the pGreenII 62-SK and pGreenII 0800-LUC vector (Hellens et al., 2005) using Seamless Cloning and Assembly Kit (Novoprotein, China), respectively. *N. benthamiana* were grown in a light incubator until four to six leaf stage when infiltration with *Agrobacterium tumefaciens* GV3101 can be performed (Yin et al., 2010). The enzyme activities of LUC and REN were determined using a previously reported protocol (Palapol et al., 2009). The measurements were performed using a luminometer Tecan Infinite M200 (Männedorf, Switzerland) from at least four biological replicates for each assay. The promoter activities were expressed as the ratios of the activities of these promoters: luciferase (LUC) to 35S: Renilla (REN).

### BiFC Assay

To verify the interaction between MaAN2 and AtTT8 *in vivo*, a BiFC assay was conducted in *A. thaliana* mesophyll protoplasts by the polyethylene glycol-mediated method. The ORF of *MaAN2* was inserted into the pSPYNE (R) 173 vector, and the coding sequence of AtTT8 without the stop codon was inserted into pSPYCE (M) (Waadt et al., 2008). The primers are listed in Supplementary Table S6. In brief, MaAN2 and AtTT8 were located adjacent to the N-terminal and C-terminal fragments of YFP, respectively. The YFP fluorescence was visualized 16 h after transformation using a confocal microscope (Olympus, Japan). The pSPYNE/MaAN2 plus pSPYCE and pSPYNE plus pSPYCE/AtTT8 were used as negative controls. Images were analyzed using the FV1000 Viewer software.

### Tobacco Stable Transformation

The function of *MaAN2* was determined by constructing a pCambia1304 vector containing the ORF of *MaAN2*. Tobacco leaf disk transformation was conducted using the previously described protocol (Horsch et al., 1985). The OE-*MaAN2* transgenic lines showing obvious color changes in leaves and flowers were used for further analysis.

### Anthocyanin Content Measurement in Grape Hyacinth Petals and Transgenic Tobacco Leaves and Flowers

The total anthocyanin content in grape hyacinth petals and transgenic tobacco leaves and flowers was determined as previously described (Mancinelli et al., 1991; Huang et al., 2016a). The petals of grape hyacinth were freeze-dried for 72 h, ground to powder, and then extracted with acidic methanolic solution (methanol, H_2_O, formic acid, and trifluoroacetic acid, 70:27:2:1, v/v) (Gao and Mazza, 1995; Hashimoto et al., 2000). The fresh leaves and petals of tobacco were ground in liquid nitrogen and then extracted. The extraction and measurement of all samples were followed by the protocol described (Huang et al., 2016a) using a UV-Visible Spectrophotometer (UV2600, Shimadzu). The subtracted absorbance was calculated as A530 (peak absorption of anthocyanins) – 0.25 × A657 (maximum absorption of chlorophyll-degradation products). The total anthocyanin content of grape hyacinth flowers and the tobacco leaves and flowers was computed as the ratio of the subtracted absorbance to dry weight and fresh weight, respectively.

In order to analyze the anthocyanin composition of tobacco leaves and flowers, we used the above-mentioned solutions for performing the reverse HPLC method. The chromatographic analysis was conducted using an Agilent 1100 series HPLC system (TC-C18 column, 5 μm, 4.6 mm × 250 mm) with the detection wavelength at 530 nm and the column was maintained at 30°C. The eluent was aqueous solution A (0.1% formic acid in water) and organic solvent B (acetonitrile). The gradient elution program was modified as described previously (Xie et al., 2006): 0 min, 10% B; 15 min, 17% B; 20 min, 23% B; 25 min, 23% B; and 30 min, 10% B; the eluent flow rate was 1.0 ml/min with 10 μl injection volume.

## Author Contributions

YL conceived and designed the research. KC and HL conducted experiments. KC drafted the manuscript. QL critically revised the manuscript. All authors read and approved the manuscript.

## Conflict of Interest Statement

The authors declare that the research was conducted in the absence of any commercial or financial relationships that could be construed as a potential conflict of interest.

## Footnotes

1 http://www.arabidopsis.org/index.jsp

2 http://bioinformatics.psb.ugent.be/webtools/plantcare/html/

## Abbreviations

AbA: aureobasidin A
BIFC: bimolecular fluorescence complementation
bHLH: basic helix–loop–helix
CK: the empty vector transgenic tobaccos
EBGs and LBGs: early and late biosynthetic genes
GFP: green fluorescent protein
HPLC: high-performance liquid chromatography
OE*-MaAN2*,: overexpressing *MaAN2* transgenic tobaccos
ORF: open reading frame
TFs: transcription factors
YFP: yellow fluorescent protein.
