# Corrigendum: Ectopic Expression of the Grape Hyacinth (*Muscari armeniacum*) R2R3-MYB Transcription Factor Gene, *MaAN2*, Induces Anthocyanin Accumulation in Tobacco

**DOI:** 10.3389/fpls.2017.01722

**Published:** 2017-09-29

**Authors:** Kaili Chen, Hongli Liu, Qian Lou, Yali Liu

**Affiliations:** ^1^College of Landscape Architecture and Arts, Northwest A&F University, Yangling, China; ^2^State Key Laboratory of Crop Stress Biology in Arid Areas, Northwest A&F University, Yangling, China; ^3^College of Horticulture, Northwest A&F University, Yangling, China

**Keywords:** flower color, monocots, grape hyacinth, R2R3-MYB transcription factor, anthocyanin biosynthesis

There is an error in the Acknowledgments section. The correct number for National Natural Science Foundation of China is 31471905.

In the original article, there was an omission in Figure 5 as published. There were 3 missing images for chloroplast autofluorescence. The corrected Figure 5 appears below.

**Figure 5:**
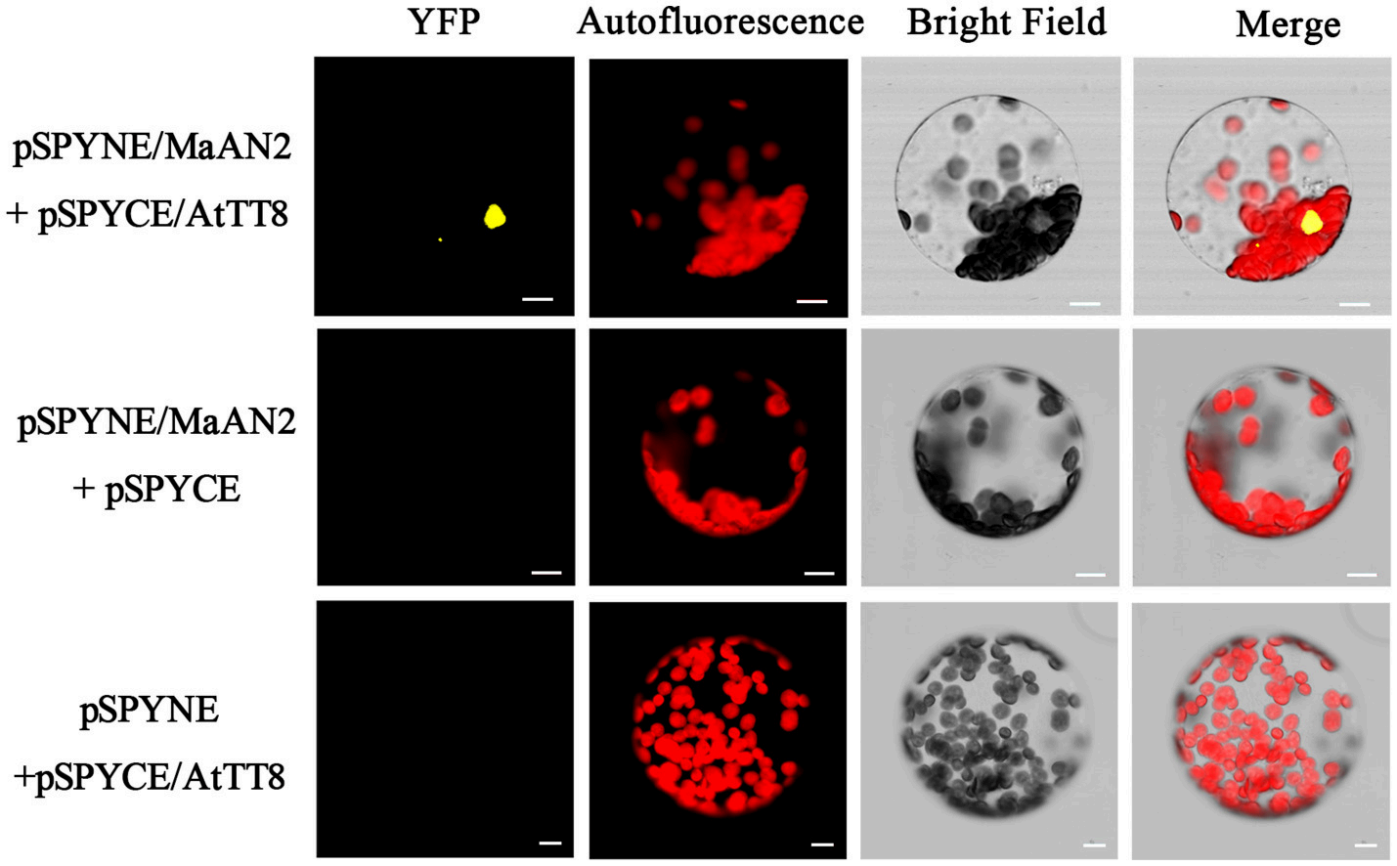
Bimolecular fluorescence complementation of MaAN2 and AtTT8 interaction in *A. thaliana* mesophyll protoplasts. YFP: fluorescence of YFP; Autofluorescence: chloroplast autofluorescence; Merge is merged with chloroplast autofluorescence, YFP fluorescence, and bright field images. Bars, 10 μm.

In the original article, there was an omission and error in the legend for Figure 5 as published. The missing information was “Autofluorescence: chloroplast autofluorescence,” and “Merge is digital image merged with bright field and fluorescent images.” should be changed into “Merge is merged with chloroplast autofluorescence, YFP fluorescence, and bright field images.” The correct legend appears below.

The authors apologize for these errors and state that this does not change the scientific conclusions of the article in any way.

## Conflict of interest statement

The authors declare that the research was conducted in the absence of any commercial or financial relationships that could be construed as a potential conflict of interest.

