# Supplementary material for: Ectopic Expression of the Grape Hyacinth (Muscari armeniacum) R2R3-MYB Transcription Factor Gene, MaAN2, Induces Anthocyanin Accumulation in Tobacco
Source: Front Plant Sci. 2017 Jun 8;8:965. doi: 10.3389/fpls.2017.00965 (PMC5462982; doi:10.3389/fpls.2017.00965)
Supplement: Supplementary file 1 [file Presentation_1.PDF]

Supplementary Material

Ectopic Expression of the Grape Hyacinth (*Muscari armeniacum*) R2R3-MYB Transcription Factor Gene, *MaAN2*, Induces Anthocyanin Accumulation in Tobacco

Kaili Chen[a], Hongli Liu[a], Qian Lou[*], Yali Liu[*]

[a]These authors contributed equally to this work.

*Correspondence

Qian Lou

louqian@nwsuaf.edu.cn

Yali Liu

lyl6151@126.com

1 Supplementary Data

All primers used in this study, including **Supplementary Tables S1 to S6**.

2 Supplementary Figures and Tables

2.1 Supplementary Figures

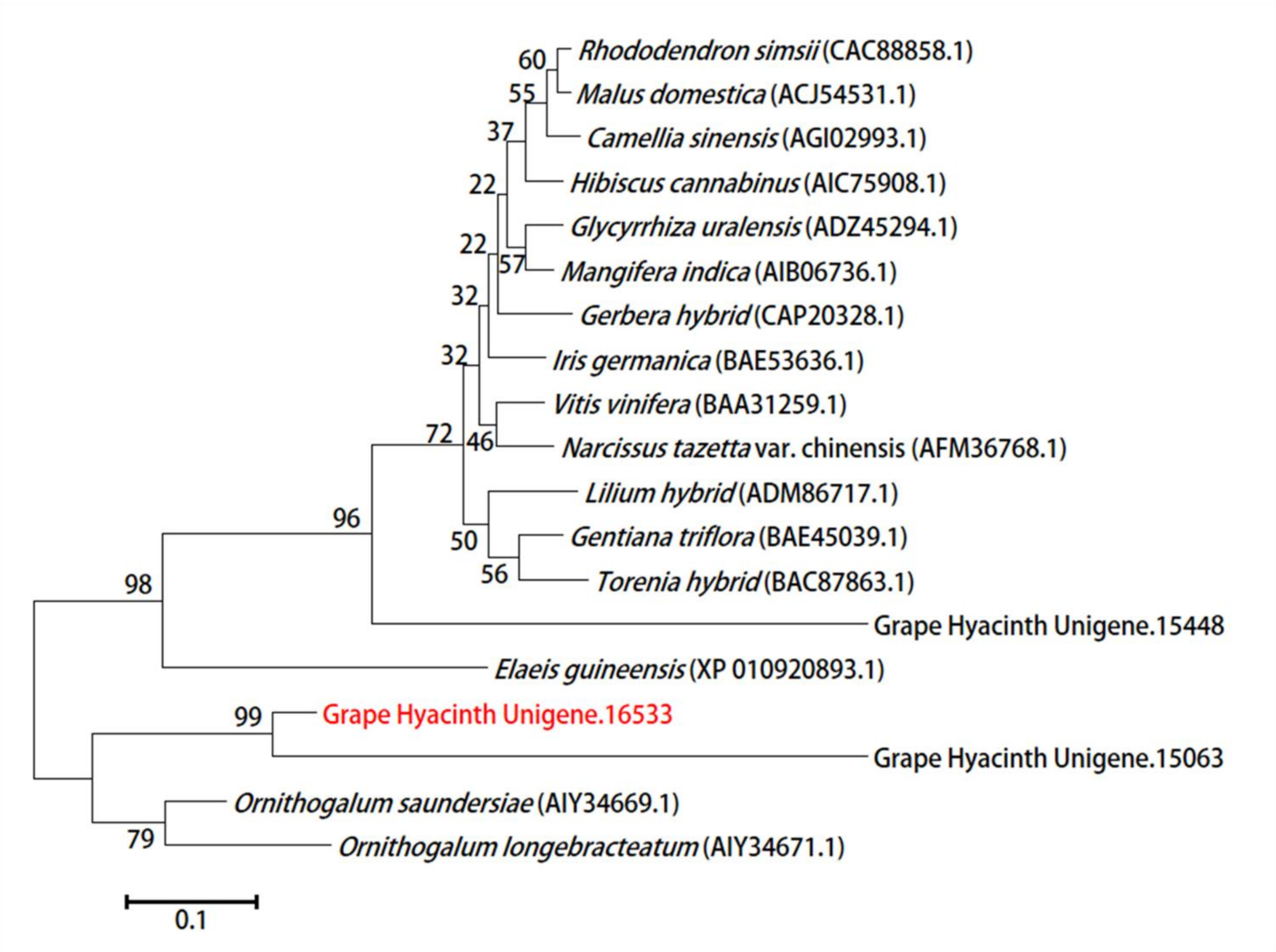

Supplementary Figure 1 Phylogenetic tree of candidate chalcone synthase from *M. armeniacum* flower transcriptome and its putative orthologs. The Grape Hyacinth Unigene.16533 used in this study was marked by the red color, which showed the highest expression in *M. armeniacum* flower compared with other candidate Unigenes (15488 and 15063). The putative protein of Grape Hyacinth Unigene.16533 was close to that of *Ornithogalum saundersiae*. The numbers in the brackets are the NCBI Genbank accession numbers of *CHS* genes in different species. The maximum-likelihood phylogenetic tree was generated using MEGA 6.0 software. Numbers next to the nodes indicate the bootstrap values from 1000 replications. The bar indicates a genetic distance of 0.1.

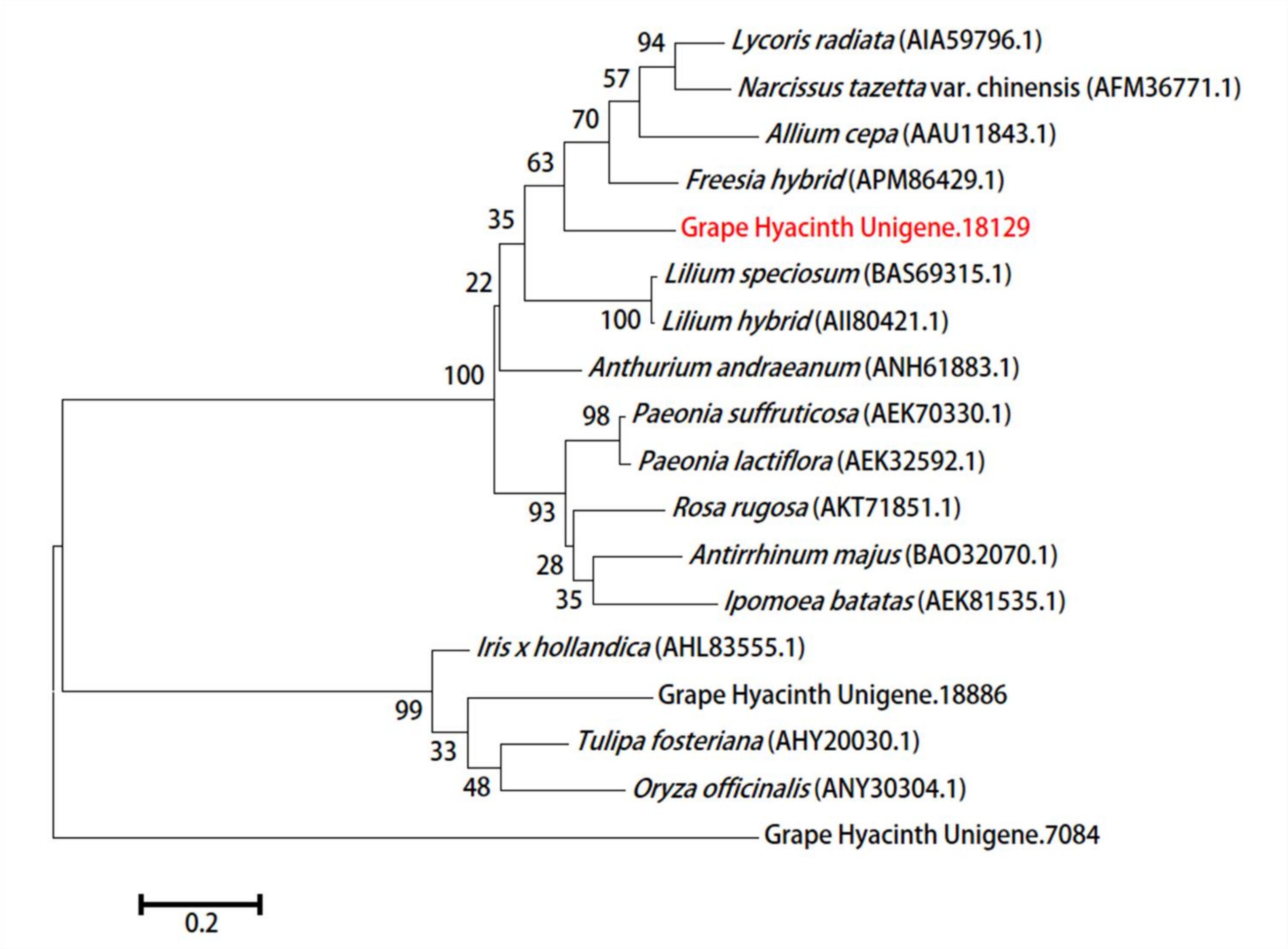

Supplementary Figure 2 Phylogenetic tree of candidate chalcone isomerase from *M. armeniacum* flower transcriptome and its putative orthologs. The Grape Hyacinth Unigene.18129 used in this study was marked by the red color, which showed the highest expression in *M. armeniacum* flower than other candidate Unigenes (18886 and 7084). The putative protein of Grape Hyacinth Unigene.18129 was close to that of *Freesia hybrid*. The numbers in the brackets are the NCBI Genebank accession numbers for the seqences of different species. The maximum-likelihood phylogenetic tree was generated using MEGA 6.0 software. Numbers next to the nodes indicate the bootstrap values from 1000 replications. The bar indicates a genetic distance of 0.2.

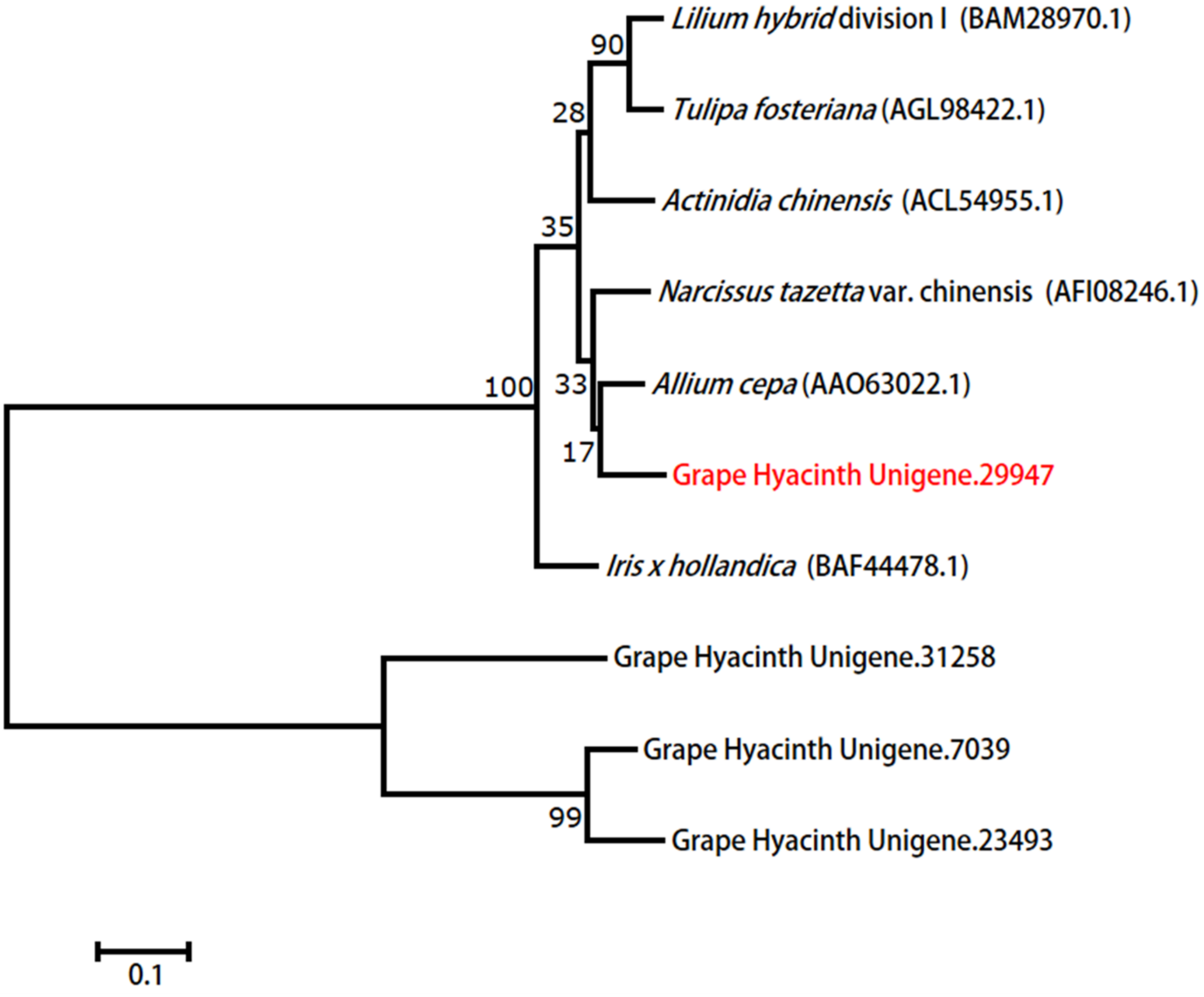

Supplementary Figure 3 Phylogenetic tree of candidate flavanone 3-hydroxylase from *M. armeniacum* flower transcriptome and its putative orthologs. The Grape Hyacinth Unigene.29947 used in this study was marked by the red color, which showed the higher expression in *M. armeniacum* flower than other candidate Unigenes (31258, 7039, and 23493). The putative protein of Grape Hyacinth Unigene.29947 was more close to that of *Allium cepa*. The numbers in the brackets are the NCBI Genebank accession numbers for the seqences of different species. The maximum-likelihood phylogenetic tree was generated using MEGA 6.0 software. Numbers next to the nodes indicate the bootstrap values from 1000 replications. The bar indicates a genetic distance of 0.1.

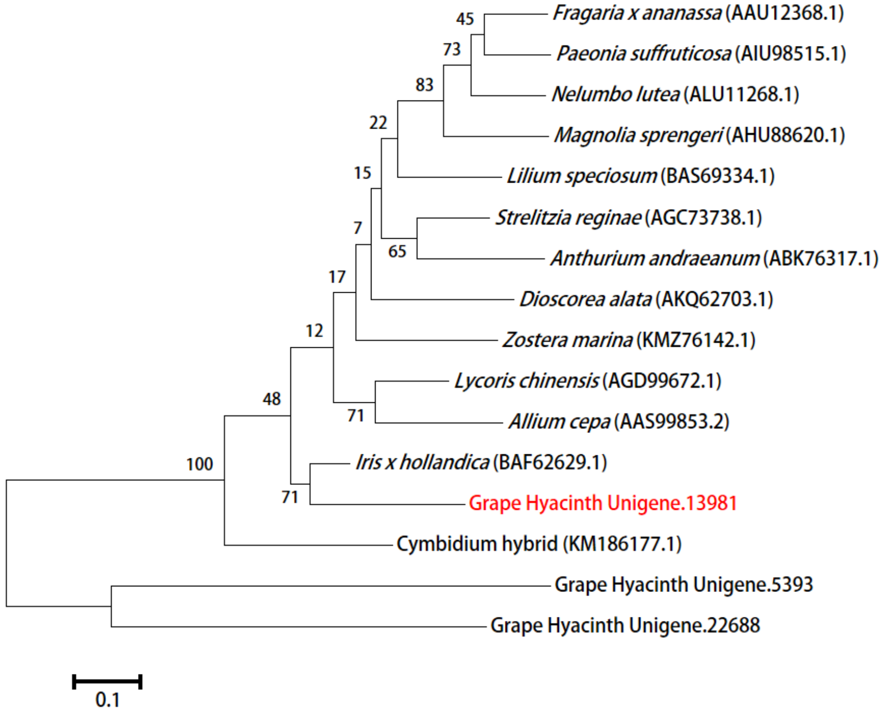

Supplementary Figure 4 Phylogenetic tree of candidate anthocyanidin synthase from *M. armeniacum* flower transcriptome and its putative orthologs. The Grape Hyacinth Unigene.13981 used in this study was marked by the red color, which showed the higher expression in *M. armeniacum* flower than other candidate Unigenes (5393 and 22688). The putative protein of Grape Hyacinth Unigene.13981 was more close to that of *Iris* × *hollandiaca*. The numbers in the brackets are the NCBI Genebank accession numbers for the seqences of different species. The maximum-likelihood phylogenetic tree was generated using MEGA 6.0 software. Numbers next to the nodes indicate the bootstrap values from 1000 replications. The bar indicates a genetic distance of 0.1.

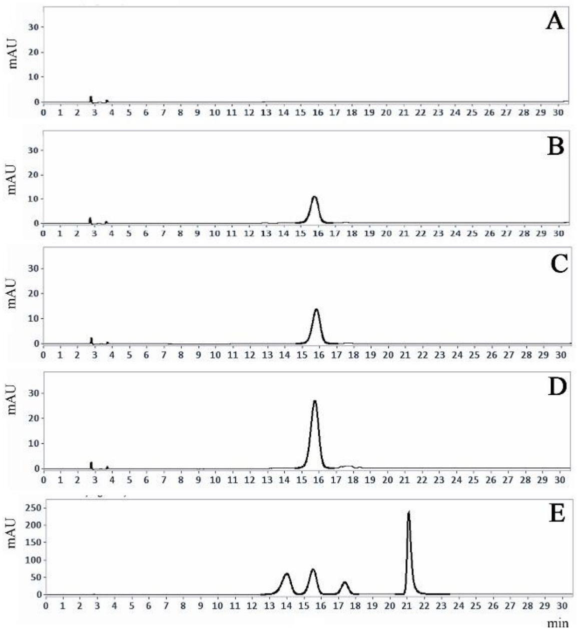

Supplementary Figure 5 The HPLC analysis of the anthocyanin extracts from the leaves and petals of tobacco transformed with an empty vector or *OE-MaAN2*. The chromatograms at 530 nm from the empty vector transgenic leaves **(A)** and petals **(B)** as well as from *OE-MaAN2* tobacco leaves **(C)** and flowers **(D)**. Peaks in diagrams A–D correspond to cyanidin-3-ruticoside, which is the major anthocyanin accumulated in the petals of common tobacco plants. The bottom chromatogram **(E)** shows the standards at 530 nm: delphinidin-3-ruticoside, cyanidin-3-ruticoside, pelargonidin-3-ruticoside, and cyanidin from left to right.
